# Phytosterols Alleviate Hyperlipidemia by Regulating Gut Microbiota and Cholesterol Metabolism in Mice

**DOI:** 10.1155/2023/6409385

**Published:** 2023-04-26

**Authors:** Wei-Jie Lv, Jie-Yi Huang, Jin Lin, Yi-Mu Ma, Shi-Qi He, Ying-Wen Zhang, Tian-Ze Wang, Ke Cheng, Ying Xiong, Feng-Gang Sun, Zhong-Chao Pan, Jing-Bo Sun, Wei Mao, Shi-Ning Guo

**Affiliations:** ^1^College of Veterinary Medicine, South China Agricultural University, State Key Laboratory of Dampness Syndrome of Chinese Medicine, Guangzhou, China; ^2^The Second Affiliated Hospital of Guangzhou University of Chinese Medicine, State Key Laboratory of Dampness Syndrome of Chinese Medicine, Guangzhou, China

## Abstract

Phytosterols (PS) have been shown to regulate cholesterol metabolism and alleviate hyperlipidemia (HLP), but the mechanism is still unclear. In this study, we investigated the mechanism by which PS regulates cholesterol metabolism in high-fat diet (HFD) mice. The results showed that PS treatment reduced the accumulation of total cholesterol (TC), triglycerides (TG), and low-density lipoprotein cholesterol (LDL-C) in the serum of HFD mice, while increasing the serum levels of high-density lipoprotein cholesterol (HDL-C). Compared with HFD mice, PS not only increased the antioxidant activity of the liver but also regulated the mRNA expression levels of enzymes and receptors related to cholesterol metabolism. The hypolipidemic effect of PS was abolished by antibiotic (Abx) intervention and reproduced by fecal transplantation (FMT) intervention. The results of 16S rRNA sequencing analysis showed that PS modulated the gut microbiota of mice. PS reduced the relative abundance of Lactobacillus and other bile salt hydrolase- (BSH-) producing gut microbiota in HFD mice, which are potentially related to cholesterol metabolism. These findings partially explain the mechanisms by which PS regulates cholesterol metabolism. This implies that regulation of the gut microbiota would be a potential target for the treatment of HLP.

## 1. Introduction

Since the 21st century, with the change of people's diet and lifestyle, obesity has gradually replaced malnutrition and infectious diseases as one of the most serious global epidemics causing medical problems [1]. High-fat diet- (HFD-) induced obesity often causes hyperlipidemia (HLP), which is a relatively common disease caused by abnormal lipid metabolism. HLP could trigger fatty liver and coronary heart disease, atherosclerosis, and other cardiovascular and cerebrovascular diseases, which seriously threaten physical health of human beings [2]. The mechanism of HLP is related to the abnormal transportation and metabolism of adipose that makes one or more lipids in serum to be higher than normal, which is characterized by increased total cholesterol (TC), triglycerides (TG), and low-density lipoprotein cholesterol (LDL-C) and decreased high-density lipoprotein cholesterol (HDL-C) in serum [3]. Currently, statins are the most widely used and effective drugs in the clinical treatment of HLP, which can reduce intracellular cholesterol synthesis by competitively inhibiting hydroxy-3-methylglutaryl-coenzyme A reductase activity [4]. Nevertheless, the use of statins has its limitations and side effects such as hepatotoxicity, rhabdomyolysis, and skeletal muscle damage [5–7]. Therefore, new interventions or drugs need to be developed to regulate lipid metabolism and mitigate the harmful effects of HFD.

**Figure 1:**
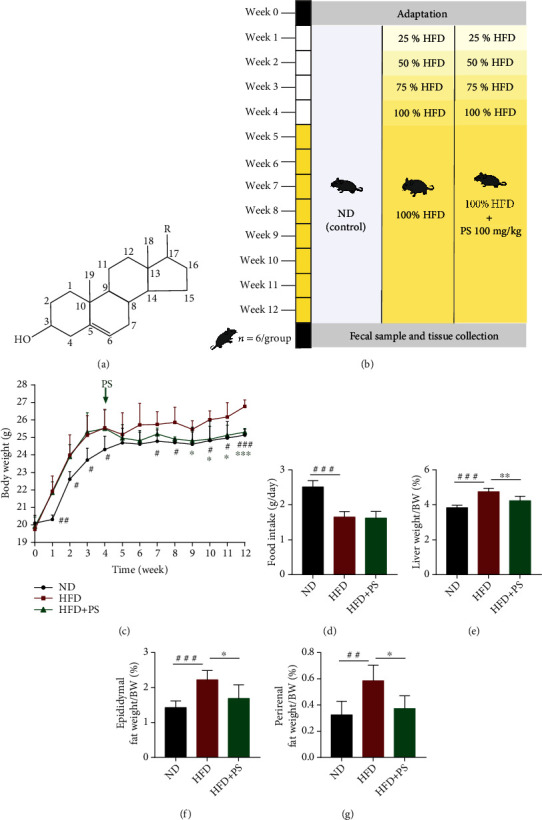
PS relieved HFD-induced fat accumulation in mice. (a) Chemical structure of PS. (b) Experiment design. (c) Body weight and (d) food intake of ND, HFD, and HFD+PS mice (*n* = 6 per group). (e–g) Liver, perirenal fat, and epididymal fat/bodyweight ratio in each group of mice (*n* = 5 per group). The data were expressed as mean ± SD. ^#^*P* < 0.05, ^##^*P* < 0.01, and ^###^*P* < 0.001 vs. ND groups; ^∗^*P* < 0.05, ^∗∗^*P* < 0.01, and ^∗∗∗^*P* < 0.001 vs. HFD groups.

Phytosterols (PS), a class of plant-derived steroids that contain the same fused four-ring core structure (Figure 1(a)) have a similar chemical structure to cholesterol [8]. PS mainly exists in all kinds of vegetable oils which is an essential component to stabilize plant cell membranes, as well as having various physiological functions such as hypolipidemic, antioxidant, anti-inflammatory, antitumor, and immunomodulatory [9, 10]. PS mainly include *β*-sitosterol, stigmasterol, campesterol, and brassicasterol [11]. The most prominent component of PS, *β*-sitosterol, is widely studied and used, and it has been shown to be effective not only in lowering blood lipids but also in relieving colon inflammation and antianxiety [12, 13]. Stigmasterol reduces serum cholesterol levels by inhibiting hepatic synthesis of cholesterol and intestinal absorption of cholesterol in rats [14]. Campesterol is reported to have cholesterol lowering, anticarcinogenic, and antiangiogenic effects [15]. Since PS are generally compounds obtained by extraction from soybean oil and other vegetable oils, they are mostly applied in the form of mixtures. Previous studies have shown that PS intervention modulates lipid metabolism and reduces oxidative damage in the brain of diabetic mice, regulates BA metabolism, and downregulates serum TC and LDL-C concentrations by 5-15%, and the role of PS in regulating gut microbiota has also been reported [16, 17]. Hence, PS are recommended to be added to such common foods as margarine, cheese, chocolate, milk, and yogurt to lower the concentration of plasma cholesterol [18, 19].

Although the benefits of PS affecting cholesterol metabolism have been reported constantly, the mechanism is still unclear. In this study, we used the HFD-induced HLP model to investigate the role of PS in regulating lipid metabolism. In addition, this study describes the role of PS in regulating the gut microbiota. We were attempting to explain the mechanism of PS in regulating cholesterol metabolism and the role of gut microbiota in this process.

## 2. Materials and Methods

### 2.1. Chemicals and Reagents

PS were supplied by Guangdong Weilai Biotechnology Co., Ltd (China). The purity of PS is more than 95%, *β*-sitosterol content is more than 40%, campesterol is more than 20%, and stigmasterol is more than 14% (Table S1). High-fat chow (fat provides 60% energy) was purchased from Beijing HFK Bioscience Co., Ltd (China). All antibiotics were purchased from Dalian Meilun Biotechnology Co., Ltd (China).

### 2.2. Animals and Diets

Five-week-old C57BL/6J male mice, weighing 18-20 g, were purchased from Beijing HFK Bioscience Co., Ltd. Animals were kept in the South China Agricultural University Laboratory Animal Center (SYXK 2019-0136) at a room temperature of 25 ± 2°C and relative humidity of 55 ± 5°C. All experiments were performed in accordance with animal welfare and the standards of the South China Agricultural University Experimental Animal Ethics Committee. All experiments were approved by the Ethics Committee.

### 2.3. Animal Experiment 1

After 1 week of adaptive feeding, C57BL/6J male mice were randomly divided into three groups: normal diet group (ND), high-fat diet group (HFD), and high-fat diet with phytosterol group (HFD+PS). In the first 4 weeks, mice in the ND group were fed normal chow, while mice in the HFD and HFD+PS groups were fed high-fat chow at 25%, 50%, 75%, and 100%, respectively. From the 5th week, mice in the HFD+PS group were gavaged with 100 mg/kg of PS. Mice in the ND and HFD groups were gavaged with equal amounts of saline once a day for 8 weeks. Mice were executed at the end of the experiment. Samples were collected, and data were recorded according to experimental necessity.

### 2.4. Animal Experiment 2

Twenty C57BL/6J male mice were adapted for 1 week and kept on high-fat chow for 12 weeks. At weeks 9 and 10, antibiotics (Abx, including vancomycin hydrochloride 0.5 g/L, metronidazole 1 g/L, clindamycin hydrochloride 5 g/L, neomycin sulfate 1 g/L, ampicillin 1 g/L, and kanamycin sulfate 5 g/L) were added to the drinking water of mice in the HFD+Abx+PS group for clearing the gut microbiota. After that, both groups of mice were gavaged with 100 mg/kg/d PS for 2 weeks. At the end of the experiment, mice were executed, and tissues were collected.

### 2.5. Animal Experiment 3

Ten C57BL/6J male mice were adaptation housed for 1 week. After 100 mg/kg/d PS gavage treatment for 2 weeks, 150 mg fresh feces of fecal microbiota transplantation (FMT) donor mice were collected into a sterile test tube every day, mixed with 0.9 mL PBS and suspended under anaerobic conditions, and vortexed for about 4 min. The mixture was centrifuged for 5 min at 4°C, 1000 × g, and the supernatant was aspirated as FMT material, gavaged to recipient mice immediately after preparation.

Twenty C57BL/6J male mice were adapted for 1 week and kept on high-fat chow for 12 weeks. At first 2 weeks, antibiotics were added to the drinking water of mice in the HFD+Abx+FMT group to clear the gut microbiota. At the 3rd week, mice in the HFD+Abx+FMT group were conducted FMT, 0.2 mL/d for 1 week, while mice in the HFD+NS group were given with normal saline. At the end of the 12th week, animals were euthanized, and tissues were collected.

### 2.6. Oral Glucose Tolerance Test (OGTT)

Mice were fasted 12 hours in advance, and blood was collected from the tail vein using a blood collection needle. Blood glucose concentration was measured with a glucometer. Subsequently, mice were gavaged with 2.0 g/kg of glucose solution. The blood glucose concentrations of mice were measured after 30 min, 60 min, 120 min, and 180 min. The concentration curves were plotted, and the area under the curve (AUC) was calculated using GraphPad Prism 7.0 software.

### 2.7. Histology of the Liver and Fat

The liver and epididymal fat of mice were fixed in 10% formalin. Hematoxylin-eosin (H&E) staining was performed on conventional paraffin-embedded tissues (nuclei were blue and cytoplasm was red), and histological examination was performed using a light microscope at 200× visual field. Frozen liver sections were stained with oil red O solution and hematoxylin solution (lipid droplets are orange to bright red and nuclei are blue). Images were acquired for analysis at 200× visual field.

### 2.8. Enzyme-Linked Immunosorbent Assay (ELISA)

The blood was collected and centrifuged at 3000 r/min for 5 min, and then, the upper serum layer was separated and stored at -80°C. Serum levels of TC and TG were measured by ELISA kit, which was purchased from Shanghai Enzyme-linked Biotechnology Co., Ltd (Shanghai, China). Serum levels of LDL-C and HDL-C were determined using ELISA kits (CUSABIO, https://www.cusabio.com/) according to the manufacturer's instructions.

### 2.9. Real-Time Quantitative PCR (RT-qPCR)

Total tissue RNA was extracted using the RNA Isolater Total RNA Extraction Reagent kit (R401), and RNA was reverse transcribed to cDNA using the HiScript III RT SuperMix for qPCR (+gDNA wiper) reverse transcription kit (R323). The reaction system was configured and performed according to the ChamQ Universal SYBR qPCR Master Mix kit (Q711), and the relative expression of target genes was analyzed by the 2^-ΔΔCt^ method data. These kits were purchased from Nanjing Vazyme Biotech Co., Ltd (China). The primers were synthesized by Tsingke Biotechnology Co., Ltd (China). The primer sequences are listed in Table S2.

### 2.10. 16S rRNA Sequencing Analysis

DNA extraction from mouse fecal samples was performed using DNeasy PowerSoil Kit (Mo Bio/QIAGEN) according to the manufacturer's instructions. The absorbance values of DNA at 260/280 nm were measured using a fluorescence spectrophotometer to assess the concentration of sample DNA. The quality of DNA was detected by 1% agarose gel electrophoresis. Primer sequences were F: ACTCCTACGGGAGGCAGCA and R: GGACTACHVGGGTWTCTAAT. The V3-V4 region of the microbial 16S rRNA gene was amplified by PCR. Sequencing was performed by Shanghai Personal Biotechnology Co., Ltd. using Illumina MiSeq gene sequencing platform.

### 2.11. Statistical Analysis

The experimental data were statistically analyzed using GraphPad Prism 7.0 software. Data comparison between two groups was analyzed by *T*-test. Multiple data groups were compared using one-way ANOVA and Tukey's multiple comparisons to analyze the variability between groups. *P* < 0.05 were considered statistically significant. Correlation analysis was performed by the genes cloud tools (https://www.genescloud.cn).

## 3. Results

### 3.1. PS Relieved HFD-Induced Fat Accumulation in Mice

**Figure 2:**
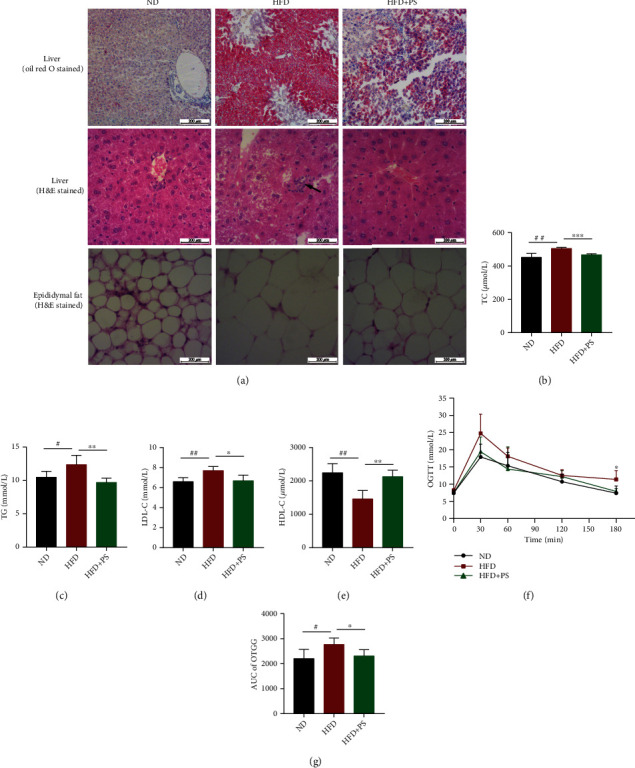
PS ameliorates disorders of glucose and lipid metabolism in mice. (a) Representative H&E staining of the liver tissue sections from each group. Scale bar: 200 *μ*m. (b–e) Serum TC, TG, LDL-C, and HDL-C concentrations (*n* = 5 per group). (f, g) Oral glucose tolerance test (OGTT) results of ND, HFD, and HFD+PS mice administrated 2.0 g/kg of glucose after 12 h of fasting and area under the curve (AUC) analysis of three different groups of mice (*n* = 4 per group). The data were expressed as mean ± SD. ^#^*P* < 0.05, ^##^*P* < 0.01, and ^###^*P* < 0.001 vs. ND groups; ^∗^*P* < 0.05, ^∗∗^*P* < 0.01, and ^∗∗∗^*P* < 0.001 vs. HFD groups.

According to the experimental design of the animal protocol, we monitored the body weight of each group of mice weekly (Figure 1(b)). Compared to the HFD mice, the body weight of the HFD+PS mice was significantly lower after 8 weeks of PS treatment (Figure 1(c)). Food intake of HFD mice and HFD+PS mice, however, was similar (Figure 1(d)). In addition, we measured the weight of the liver, perirenal fat, and epididymal fat and observed histopathological changes in lipid accumulation and inflammatory changes in the liver. The results showed a significant increase in the organ-to-body weight ratio of the liver (Figure 1(e)), perirenal fat (Figure 1(f)), and epididymal fat (Figure 1(g)) in HFD mice compared to ND mice and a significant decrease in the ratio in HFD+PS mice compared to HFD mice. The results of oil red O stained and H&E-stained sections of the liver showed that PS alleviated the hepatic steatosis and inflammatory changes in HFD mice (Figure 2(a)). H&E-stained sections of epididymal fat showed that PS reduced the adipocyte area in HFD mice (Figure 2(a)). In conclusion, PS intervention reduced HFD-induced fat accumulation in mice.

### 3.2. PS Ameliorates Disorders of Glucose and Lipid Metabolism in Mice

Next, to determine whether PS ameliorated glucolipid metabolism, we measured the serum concentrations of TG, TC, LDL-C, and HDL-C in mice, which are considered clinical biomarkers of severe HLP [9]. The concentrations of TG, TC, and LDL-C were significantly increased, and the HDL-C was decreased in HFD mice. As expected, the concentrations of TG, TC, and LDL-C were significantly decreased, and HDL-C was increased following PS treatment (Figures 2(b)–2(e)). To explore whether PS regulated glucose tolerance of mice, we measured the blood glucose concentration at 30 min intervals during the 3 hours of oral glucose administration. Blood glucose profiles and the area under the curve (AUC) of OGTT showed that HFD mice had increased glucose AUC and impaired glucose tolerance, while HFD+PS mice had significantly improved glucose tolerance compared to HFD mice (Figures 2(f) and 2(g)).

### 3.3. PS Improves Antioxidant Activity in the Liver of HFD Mice

**Figure 3:**
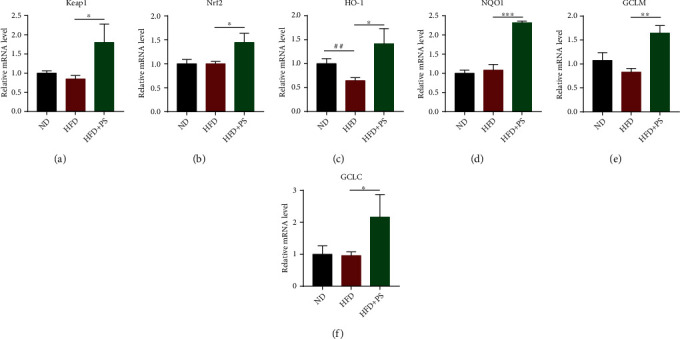
PS improves antioxidant activity in the liver of HFD mice. (a–f) The relative mRNA expression of antioxidant genes in the liver, including Keap1, Nrf2, HO-1, NQO1, GCLM, and GCLC (*n* = 3 per group). The data were expressed as mean ± SD (*n* = 3 per group). ^##^*P* < 0.01 vs. ND groups; ^∗^*P* < 0.05, ^∗∗^*P* < 0.01, and ^∗∗∗^*P* < 0.001 vs. HFD groups.

Next, we examined the levels of oxidative stress-related mRNA in the liver. Compared with the ND mice, HFD significantly decreased the mRNA levels of heme oxygenase 1 (HO-1); compared with the HFD group mice, PS significantly increased the mRNA levels of Kelch-like ECH-associated protein 1 (Keap1), nuclear factor, erythroid 2 like 2 (Nrf2), HO-1, NAD(P)H quinone dehydrogenase 1 (NQO1), glutamate-cysteine ligase modifier subunit (GCLM), and glutamate-cysteine ligase catalytic subunit (GCLC) (Figures 3(a)–3(f)). These results suggested that PS not only did not induce hepatocyte damage but also greatly improved resistance to HFD-induced oxidative stress.

These results indicated that HFD significantly increased the concentrations of serum TG, TC, and LDL-C and decreased HDL-C, causing HLP and leading to impaired glucose tolerance in mice. PS treatment reversed these changes and ameliorated HLP and glucose tolerance.

### 3.4. PS Regulates the mRNA Expression of Enzymes and Receptors Related to Cholesterol Metabolism

**Figure 4:**
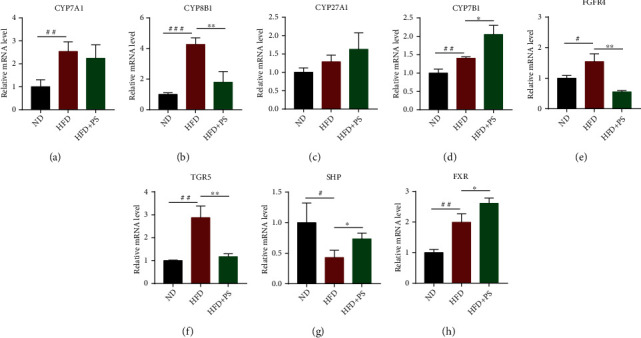
PS regulates the mRNA expression of enzymes and receptors related to cholesterol metabolism. (a–d) The relative mRNA expression of CYPs in the liver, including CYP7A1, CYP8B1, CYP7B1, and CYP27A1. (e–h) The relative mRNA expression of FGFR4, TGR5, FXR, and SHP in the liver. The data were expressed as mean ± SD (*n* = 3 per group). ^#^*P* < 0.05, ^##^*P* < 0.01, and ^###^*P* < 0.001 vs. ND groups; ^∗^*P* < 0.05 and ^∗∗^*P* < 0.01 vs. HFD groups.

To investigate the mechanism of PS to ameliorate HLP, we first measured the mRNA levels of cholesterol 7*α*-hydroxylase (CYP7A1), sterol 12*α*-hydroxylase (CYP8B1), oxysterol 7*α*-hydroxylase (CYP7B1), and sterol 27-hydroxylase (CYP27A1) and the bile acid (BA) synthesis rate-limiting enzyme related to cholesterol metabolism. It showed that compared with ND mice, the expressions of CYP7A1 and CYP8B1 were strongly increased in HFD mice, and the expressions of CYP27A1 and CYP7B1 also showed an increasing trend. PS intervention caused a significant decrease in the relative mRNA expression of CYP8B1 and a downward trend of CYP7A1 in HFD mice. CYP7B1 was significantly upregulated, and CYP27A1 was trended to increase in HFD+PS mice (Figures 4(a)–4(d)).

Next, we also analyzed the relative mRNA expression of receptors related to cholesterol metabolism in the liver. The results showed that HFD caused an increase in the relative mRNA expression of fibroblast growth factor receptor 4 (FGFR4), Takeda G-protein-coupled receptor 5 (TGR5), and Farnesoid X receptor (FXR) and a decrease in the relative mRNA expression of small heterodimer protein (SHP) in mice. In HFD mice, PS treatment resulted in a significant decrease in the relative mRNA expression of TGR5 and FGFR4 and a significant increase in the mRNA levels of FXR and SHP in the liver (Figures 4(e)–4(h)).

As mentioned above, PS inhibited the classical pathway of hepatic cholesterol metabolism and promoted the expression of rate-limiting enzymes of alternative pathways. Meanwhile, PS inhibited TGR5 and FGFR4 and promoted the expression of FXR and SHP receptors.

### 3.5. The Effect of PS in Relieving HLP Depends on the Gut Microbiota

**Figure 5:**
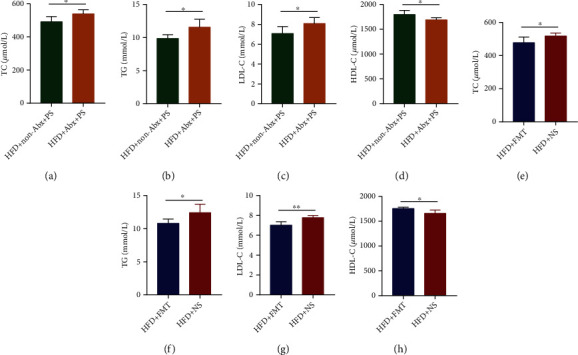
The effect of PS in relieving HLP depends on the gut microbiota. (a–d) Serum TC, TG, LDL-C, and HDL-C concentrations in HFD+Abx+PS and HFD+non-Abx+PS mice. (e–h) Serum TC, TG, LDL-C, and HDL-C concentrations in HFD+NS and HFD+FMT mice. The data were expressed as mean ± SD (*n* = 5 per group). ^∗^*P* < 0.05 and ^∗∗^*P* < 0.01.

Previous studies have shown that the gut microbiota and its metabolites are closely related to cholesterol metabolism [20, 21]. To verify whether the hypolipidemic effect of PS is related to the gut microbiota, we treated mice with Abx and FMT. The results showed that compared with HFD+non-Abx+PS mice, TG, TC, and LDL-C were significantly higher, and HDL-C was lower in HFD+Abx+PS mice (Figures 5(a)–5(d)). That means the hypolipidemic effect of PS was eliminated. In addition, we treated FMT donor mice with PS and administered FMT to Abx-pretreated HFD mice. Compared with HFD+NS mice, HFD+FMT mice showed significantly lower TG, TC, and LDL-C and significantly higher HDL-C (Figures 5(e)–5(h)), and their HLP was considerably alleviated. In conclusion, the effect of PS in relieving HLP is strongly related to the gut microbiota.

### 3.6. PS Alters the Structure of the Gut Microbiota in Mice

**Figure 6:**
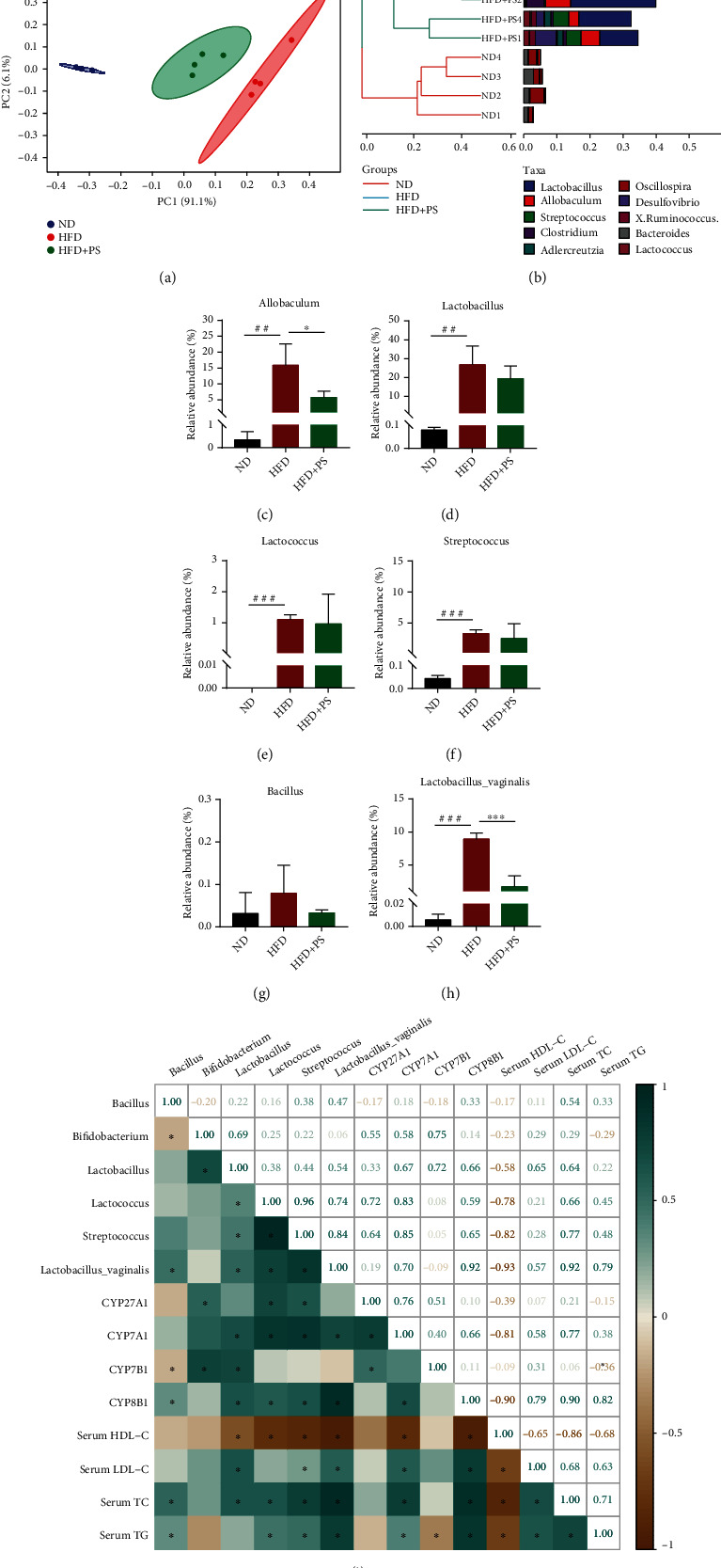
PS alters the structure of the intestinal microbiota in mice. (a) Principal component analysis (PCA) plot. (b) Hierarchical clustering analysis. (c–g) The relative abundance of gut microbiota at the genus level, including Allobaculum, Lactobacillus, Lactococcus, Streptococcus, and Bacillus (*n* = 4 per group). (h) The relative abundance of Lactobacillus vaginalis. (i) Pearson's correlation coefficients among gut microbiota, TC, TG, LDL-C, HDL-C, and CYPs. The data were expressed as mean ± SD. ^##^*P* < 0.01 and ^###^*P* < 0.001 vs. ND groups; ^∗^*P* < 0.05 and ^∗∗∗^*P* < 0.001 vs. HFD groups.

Firstly, the results of principal component analysis (PCA) of gut microbiota showed that there were different clusters of gut microbial species abundance in the ND, HFD, and HFD+PS groups (Figure 6(a)). Also, hierarchical clustering analysis showed that PS treatment altered the gut microbiota of HFD mice (Figure 6(b)). Analysis of microbial community composition at the genus level showed that the relative abundance of Allobaculum was reduced in HFD+PS mice compared to HFD mice (Figure 6(c)), and Lactobacillus, Lactococcus, Streptococcus, and Bacillus showed a decreasing trend (Figures 6(d)–6(g)). Remarkably, at the species level, PS treatment led to a decrease in Lactobacillus vaginalis in HFD mice (Figure 6(h)). Pearson's correlation coefficients showed that, in general, the relative abundance of these bile salt hydrolase- (BSH-) producing gut microbiota was significantly correlated with the concentrations of TG, TC, LDL-C, and HDL-C and the expression of cytochrome P450s (CYPs) (Figure 6(i)). These results suggest that the hypolipidemic effect of PS is related to the improvement of the structure of the gut microbiota in mice.

## 4. Discussion

HLP is a common and prevalent disease that occurs in obese or overweight people, and it is often complicated with diabetes, hypertension, and coronary heart disease [22, 23]. The causes of HLP vary from one type to another, with excess dietary fat, obesity, and prolonged heavy alcohol consumption being its common causes [24]. Worldwide, the prevalence and incidence of HLP have been increasing rapidly. Studies have shown that there are significant physiological differences between males and females in not only estrogen but also proteins involved in cholesterol homeostasis, which explains why premenopausal females are better able to maintain cholesterol homeostasis and resist HLP [25, 26]. Males are more susceptible to HLP, which is the reason we chose male mice as experimental animals. Statins are potent agents targeting HLP, significantly lowering TC levels, as well as lowering TC and LDL-C levels, and raising HDL-C [27]. However, it mainly has side effects such as elevated transaminases and myopathy, including myalgia, myositis, and transverse muscle melting, which predispose to poor prognosis [6, 28]. PS are widely used in medical research because of their widespread presence in nature and their powerful properties [29]. Multiple studies have shown that PS significantly reduce blood cholesterol concentrations which has important clinical value in the management of obesity, HLP, and diabetes [30–32]. In our study, PS treatment alleviated HLP in HFD mice with a significant decrease in serum TG, TC, and LDL-C levels and a significant increase in HDL-C levels. These changes are consistent with the reported hypolipidemic effect of PS [33]. In addition, we tested the effect of PS on hepatic antioxidant function. In our experiments, PS elevated the relative mRNA expression of Keap1, Nrf2, HO-1, NQO1, GCLM, and GCLC in HFD mice. This study showed that PS treatment not only did not cause liver damage in HFD mice but also enhanced the antioxidant effect of the liver.

We hypothesized that the hypolipidemic effect of PS is related to the pathway of cholesterol metabolism in the liver. BA metabolism is an important route of cholesterol excretion. About one-third of the cholesterol catabolism in the body is achieved by BA synthesis [34]. The enterohepatic circulation of BA plays a central role in nutrient absorption, distribution, metabolic regulation, and homeostasis [34]. BAs are produced by a series of CYPs in the liver that catalyze the oxidation of cholesterol through two biosynthetic pathways, called classical pathway and alternative pathway [35]. In the classical pathway, CYP7A1 rate-limiting enzyme mediates the production of cholic acid (CA), and CYP8B1-/- mice failed to produce CA and had an increased expression of CYP7A1 and an expanded BA pool [36]. In the alternative pathway, CYP27A1 rate-limiting enzyme mediates the catalytic formation of 27-hydroxycholesterol, which is then further catalyzed by CYP7B1 to produce chenodeoxycholic acid (CDCA) [37]. CD and CDCA are both primary BAs synthesized directly by hepatocytes using cholesterol as a raw material. The primary BAs produced are then combined with taurine and glycine to form conjugated BA, which are actively transported into the bile via the bile salt output pump, stored in the gallbladder, and subsequently released into the duodenum to function [38]. In our experiments, PS intervention downregulated CYP7A1 and CYP8B1 enzymes in the classical pathway of BA synthesis in HFD mice, whereas CYP27A1 and CYP7B1 enzymes in the alternative pathway of BA synthesis were upregulated. We hypothesize that PS improves cholesterol metabolism by regulating the expression of these rate-limiting enzymes.

According to the previous reports, signaling by FXR and FGFR4 receptors in the liver can control BA synthesis pathways in the liver, while BA transport in the enterohepatic cycle alters the composition, size, and distribution of the BA pool, which in turn can have a significant impact on BA signaling and its downstream metabolic targets [39]. FXR is activated exclusively by BA, and in the intestine, activation of FXR promotes the release of FGF15 and the signaling to target tissue receptors [40–42]. It is found that upregulation of FGF15-FGFR4 signaling accelerates the progression of nonalcoholic fatty liver disease (NAFLD) to hepatocellular carcinoma (HCC) [43]. In our study, PS downregulated hepatic FGFR4 expression, and inhibition of FGFR4 signaling has been reported to improve hepatic metabolism and is a promising option for the treatment of NAFLD and HCC [44–46]. Modulation of the liver FXR-SHP pathway can regulate cholesterol conversion and fatty acid metabolism, thus reducing the accumulation of hepatic lipids [47]. FXR regulates bile acid synthesis through inhibition of CYP7A1-negative feedback by inducing SHP [48]. PS intervention activated the FXR-SHP pathway in the liver, which is thought to inhibit the classical pathway of BA synthesis and activate the alternative pathway [49]. Besides, there is evidence that FXR leads to significant changes in intestinal fat absorption, as well as a selective reduction in lipogenesis [50]. As such, the FXR-SHP pathway may become a viable therapeutic approach to modulate BA and lipid metabolism for the treatment of HFD-induced HLP [51]. TGR5 is a specific cell surface receptor that responds directly to BA and protects the liver from BA overload by controlling bile hydrophobicity and cytokine secretion [52, 53]. TGR5 regulates the expression and activity of genes involved in BA, lipid, and carbohydrate metabolism, energy expenditure, and inflammation [54]. We suggest that the hypocholesterolemic effects of PS are mediated by reducing TGR5 and FGFR4 receptors and increasing the expression of FXR and SHP receptors in the liver, which inhibit the classical pathway of BA synthesis and promote the alternative pathway. These findings partially explain the molecular mechanisms by which PS regulates BA metabolism and suggest that alternative and classical pathways regulating BA synthesis may be potential targets for the treatment of HLP. The intervention of PS regulates the expression of CYPs through the activation or inhibition of receptors, which ultimately affects cholesterol metabolism.

As mentioned above, BA synthesis is an important pathway of cholesterol metabolism. Signaling in the hepatic-intestinal axis directly regulates the relationship between gut microbiota and BA metabolism [37, 55]. In the intestine, the structure of gut microbiota and microbial metabolism of BA have an interaction, and microbial metabolism can form a hydrophobic pool of BA and enhance BA clearance [56]. Bile salt hydrolases (BSH) hydrolyze conjugated BAs, and these reactions occur mainly in the distal small intestine and proximal colon in association with BSH-producing bacterial populations [57]. According to the reports, BSH activity was mainly derived from *Lactobacillus*, *Lactococcus*, *Streptococcus*, *Bacillus*, and *Bifidobacterium*, and the highest enzymatic activity was reported for BSH-T3 present in *Lactobacillus* [55, 58]. In contrast, reducing the relative abundance of *Lactobacillus* reduces BSH activity and leads to changes in the composition of BA, ultimately alleviating obesity [59]. Previous studies have shown that lipid metabolism can be ameliorated by regulating gut microbiota and BA metabolism in HFD mice [60]. In this study, the hypolipidemic effect of PS was found to be eliminated after Abx treatment of mice. However, after FMT, HFD mice possessed the ability to resist HLP. Therefore, we speculate that the hypolipidemic effect of PS is related to the gut microbiota. Correlation analysis showed that the relative abundance of *Lactobacillus*, *Lactococcus*, *Streptococcus*, and *Lactobacillus vaginalis* was strongly associated with hyperlipidemia; also, these genera and the expression of CYP7A1 and CYP8B1 were positively correlated, while *Bacillus* was negatively correlated with CYP7B1. Our analysis of the gut microbiota showed that PS reduced the relative abundance of *Lactobacillus* and other BSH-producing gut microbiota in HFD mice, which is probably critical for regulating BA metabolism [61, 62]. Intriguingly, PS treatment significantly reduced the relative abundance of *Lactobacillus vaginalis*. Furthermore, *Allobaculum* was thought to be positively correlated with HLP, and this was reinforced by the fact that PS intervention resulted in a significant reduction of *Allobaculum* in HFD mice [63, 64].

In previous studies, the mechanisms of cholesterol absorption inhibition by PS are mainly focused on competition for binding sites, reduction of cholesterol absorption, and interference with BA metabolism mechanisms [65, 66]. Some studies suggest that PS are not absorbed by the body, but their structural similarity to cholesterol allows them to inhibit cholesterol absorption, including recirculating endogenous biliary cholesterol [67, 68]. In other words, PS may affect BA metabolism by directly affecting the structure of the gut microbiota or by indirectly inhibiting cholesterol absorption. One hypothesis is that PS directly regulates the structure of the gut microbiota in the intestine, thereby affecting the activity of BSH and reducing the hydrolysis of conjugated BA and then BA synthesis. Another hypothesis is that PS reduces cholesterol absorption through competition with cholesterol, thereby reducing liver cholesterol metabolism and BA synthesis in HFD mice. Both hypotheses need to be tested by more in-depth experiments. Additional well-designed studies should be developed in animal models and humans to test the safety and efficacy. Nevertheless, whether the effect of PS is direct or indirect, our study illustrates the clinical relevance of PS in alleviating HLP.

## 5. Conclusions

In summary, we found that PS significantly increased the antioxidant function of the liver and alleviated HLP in mice. The mechanism was related to the regulation of cholesterol metabolism and the structure of the gut microbiota. The relative abundance of the gut microbiota is regulated when treated with PS. SHP and FXR were activated, and FGFR4 and TGR5 were inhibited, while the classical pathway of BA synthesis (CYP7A1 and CYP8B1) was inhibited, and the alternative pathway of BA synthesis (CYP27A1 and CYP7B1) was activated treated with PS. Our results partially explain the mechanism of PS treatment of HLP, which may be related to the regulating cholesterol metabolic pathways and the structure of the gut microbiota.

## Data Availability

The raw fastq files of 16S rRNA sequencing analysis in this study were deposited in the National Center for Biotechnology Information (NCBI BioProject: PRJNA855593). All data sets generated or analyzed during this study are included in the article and supplementary materials. Data are available upon reasonable request.

## Supplementary Materials

Supplementary Materials The following supporting information can be downloaded in the Supplementary Material for comprehensive analysis. Table S1: analysis of phytosterol content. Table S2: sequence of the primers used for quantitative RT-PCR assay. Graphical abstract.
