# Supplementary material for: Phytosterols Alleviate Hyperlipidemia by Regulating Gut Microbiota and Cholesterol Metabolism in Mice
Source: Oxid Med Cell Longev. 2023 Apr 26;2023:6409385. doi: 10.1155/2023/6409385 (PMC10156461; doi:10.1155/2023/6409385)
Supplement: Supplementary Materials — The following supporting information can be downloaded in the Supplementary Material for comprehensive analysis. Table S1: analysis of phytosterol content. Table S2: sequence of the primers used for quantitative RT-PCR assay. Graphical abstract. [file 6409385.f1.zip › Hindawi_Table S1 (1).docx]

**TABLE S1：**Analysis of phytosterol content.

| **SUBJECT** | **RESULT** | **LIMIT** | **UNIT** |
| --- | --- | --- | --- |
| TOTAL STEROLS(TS) | 5.2 | 5.0 MIN | % |
| BRASSICASTEROL/TS | 3.9 | 0-6 | % |
| CAMPESTEROL/TS | 28.1 | 20-30 | % |
| STIGMASTEROL/TS | 17.2 | 14-22 | % |
| β-SITOSTEROL/TS | 43.9 | 40-58 | % |
| β-SITOSTANOL/TS | 2.2 | 0-5 | % |
| CAMPESTANOL/TS | 0.6 | 0-5 | % |
| OTHER STEROLS/TS | 3.7 | 0-6 | % |

METHOD: GB/T 39995-2021

SAMPLE WEIGHT: 500 g
