# Supplementary material for: Phytosterols Alleviate Hyperlipidemia by Regulating Gut Microbiota and Cholesterol Metabolism in Mice
Source: Oxid Med Cell Longev. 2023 Apr 26;2023:6409385. doi: 10.1155/2023/6409385 (PMC10156461; doi:10.1155/2023/6409385)
Supplement: Supplementary Materials — The following supporting information can be downloaded in the Supplementary Material for comprehensive analysis. Table S1: analysis of phytosterol content. Table S2: sequence of the primers used for quantitative RT-PCR assay. Graphical abstract. [file 6409385.f1.zip › Hindawi_Table S2.docx]

**TABLE S2：** Sequence of primers used for Quantitative RT-PCR assay.

| **Gene** | **Forward primer (5′-3′)** | **Reverse primer (5′-3′)** |
| --- | --- | --- |
| β-Actin | TGCTGTCCCTGTATGCCTCTG | CTGTAGCCACGCTCGGTCA |
| Keap1 | TCCATTGAAGGCATCCACCC | GGGCAGTCGTATTTGACCCA |
| Nrf2 | CCTCTGTCACCAGCTCAAGG | TGGGCGGCGACTTTATTCTT |
| HO-1 | GCTGGTGATGGCTTCCTTGT | GCATAGACTGGGTTCTGCTTGTT |
| NQO1 | AGGACGCCTGAGCCCAGATA | CTGGAAAGGACCGTTGTCGTAC |
| GCLC | ACCTGGATGATGCCAACGAGT | CCCTAGTGAGCAGTACCACGAATA |
| GCLM | CTGTATCAGTGGGCACAGGTAA | AGAGCAGTTCTTTCGGGTCATT |
| CYP7A1 | TCCACCTTTGATGACATGGA | GAAGGTTGCAGGAATGGTGT |
| CYP8B1 | TGGCCTCTTTCACTTCTGCT | ATCCAGGTCTTGCTGCTTGT |
| CYP27A1 | GAGAGTGAATCAGGGGACCA | CTCAGGAATGGAGGGTTTCA |
| CYP7B1 | TCTCTGGGCCTCTCTAGCAA | CAGGGCTTCCATAGCTTCAG |
| FXR | AATGAGGACGACAGCGAAGG | TGCCGTGAGTTCCGTTTTCT |
| FGFR4 | TGTCTACCCACAGCAAGCAC | TGCCTCCAATACGATTCTCC |
| SHP | GCACGATCCTCTTCAACCCA | CAGAAGGGTGCCTGGAATGT |
| TGR5 | CTCATCGTCATCGCCAACCT | CAAGTGGAGAAGGAGGCAGG |
