# Supplementary figures and images for: Phytosterols Alleviate Hyperlipidemia by Regulating Gut Microbiota and Cholesterol Metabolism in Mice
Source: Oxid Med Cell Longev. 2023 Apr 26;2023:6409385. doi: 10.1155/2023/6409385 (PMC10156461; doi:10.1155/2023/6409385)

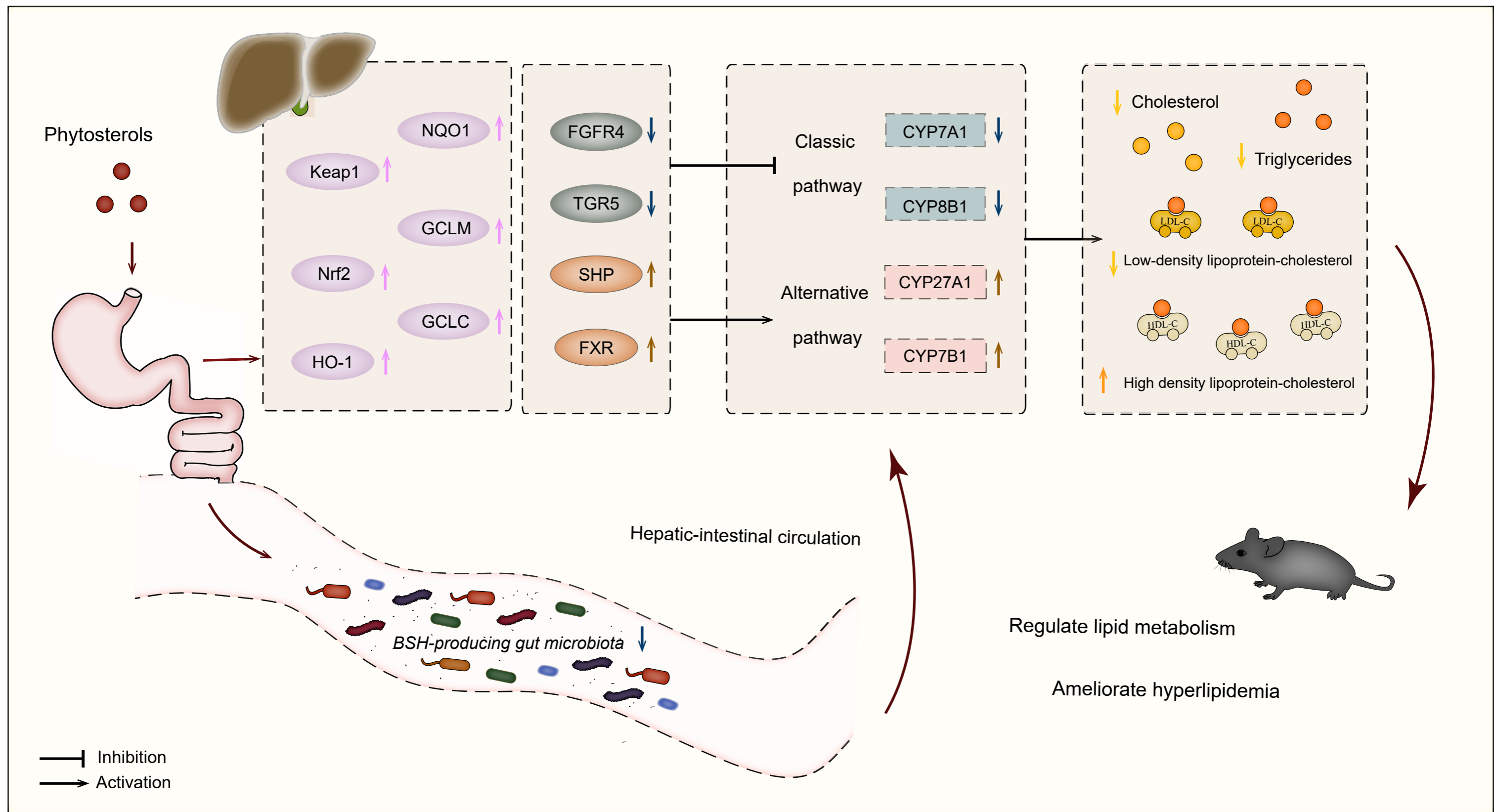

Supplement: Supplementary Materials — The following supporting information can be downloaded in the Supplementary Material for comprehensive analysis. Table S1: analysis of phytosterol content. Table S2: sequence of the primers used for quantitative RT-PCR assay. Graphical abstract. [file 6409385.f1.zip › Graphical abstract (1).pdf]
